# Off-pump Coronary Artery Bypass Grafting in Moyamoya Disease: a Case Report

**DOI:** 10.21470/1678-9741-2017-0219

**Published:** 2018

**Authors:** Elif Coşkun, Levent Altinay, Ufuk Tutun, Anıl Tekin

**Affiliations:** 1 Department of Cardiovascular Surgery, Medical Faculty, Zonguldak Bulent Ecevit University, Zonguldak, Turkey.

**Keywords:** Moyamoya Disease, Coronary Artery Bypass, Off-Pump, Coronary Artery Bypass, Coronary Stenosis/Complications/Surgery

## Abstract

Moyamoya disease is a rare, idiopathic, progressive, occlusive disease of the internal carotid artery characterized by the development of collateral vasculature in the brain base. In patients with accompanying coronary artery disease, cardiopulmonary bypass posses a potential risk for perioperative cerebral ischemic complication. Herein, we report a 53-year-old male case of Moyamoya disease and coronary artery disease who was treated with off-pump coronary artery bypass grafting.

**Table:** 

Abbreviations, acronyms & symbols	
CABG	= Coronary artery bypass grafting
DSA	= Digital subtraction angiographic
IABP	= Intra-aortic balloon pump
MCA	= Middle cerebral artery

## INTRODUCTION

Moyamoya disease is characterized by the occlusion or spontaneous bilateral stenosis of the terminal portion of the internal carotid artery^[^^1^^]^. There is a limited number of reports on extracranial vessel involvement and accompanying ischemic events in Moyamoya disease^[^^2^^]^.

We report a 53-year-old male case of Moyamoya disease having coronary ischemia treated with off-pump coronary artery bypass grafting (CABG).

## CASE REPORT

**Fig. 1:**
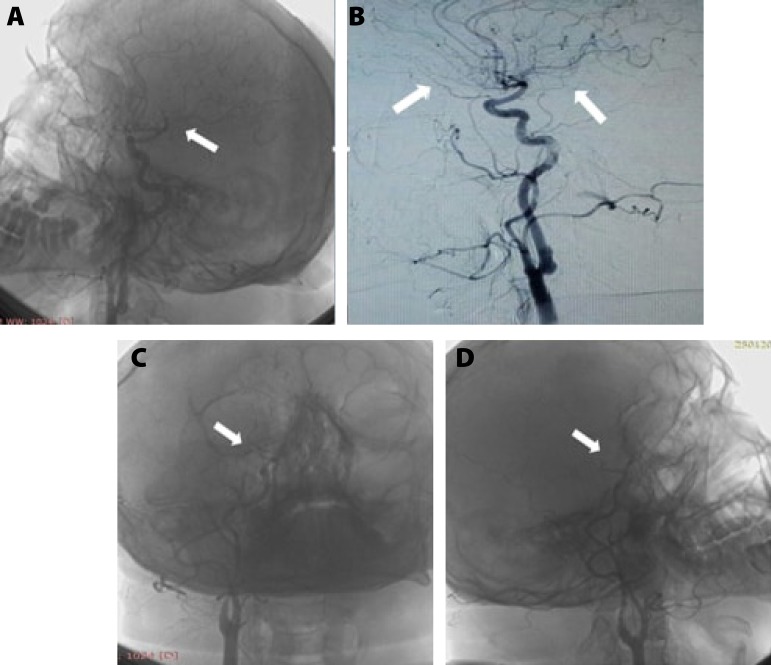
Cerebral Artery Digital Subtraction Angiography (DSA). A e B) Left internal carotid arterial injection revealed a normal middle and anterior cerebral arterial blood flow. C e D) Right internal carotid arterial injection revealed a stenosis involving the distal ophthalmic branch without any blood flow in the right middle and anterior cerebral arteries.

A 53-year-old male patient was referred to our emergency department due to a sudden-onset chest pain. In his medical history he had coronary artery disease for five years and a CABG surgery was recommended for him in another healthcare centre one year before. The stenosis in the right internal carotid artery was observed in the preoperative carotid Doppler ultrasonography. The patient was then diagnosed with Moyamoya disease based on subsequent cranial computed tomography and cerebral digital subtraction angiographic (DSA) images (Figure 1). In the DSA scans obtained from the previous institution, the stenosis of the right internal carotid artery extended to the distal ophthalmic branch was observed. Due to the low blood flow in the right middle cerebral artery (MCA), CABG was considered risky for causing cerebral hypoperfusion so their treatment plan was revised to the placement of a coronary stent into the main circumflex artery.

**Fig. 2:**
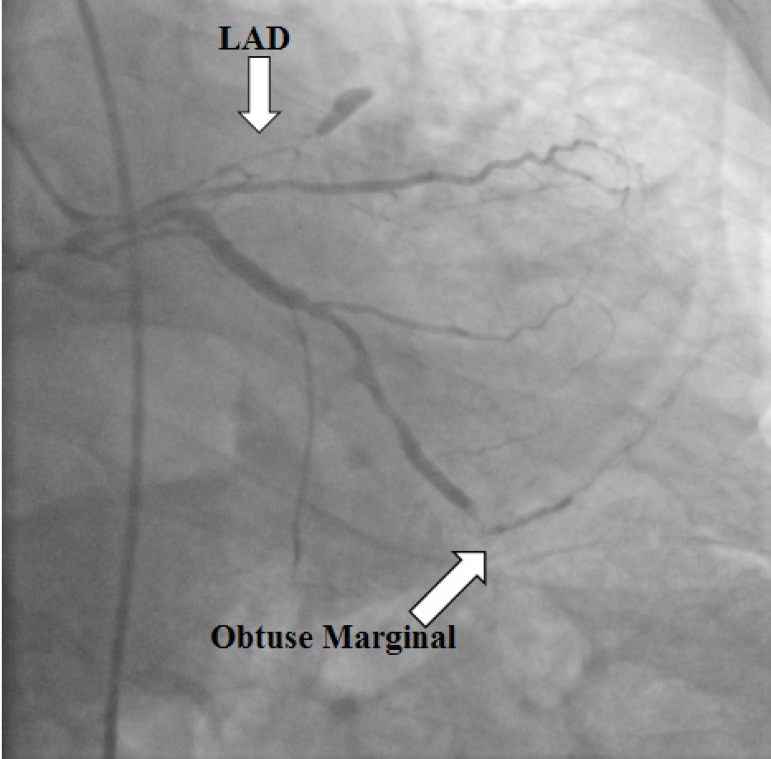
Coronary angiography showing 100% stenosis in the left anterior descending artery (LAD) and 50% stenosis in the second obtuse marginal artery, followed by 100% stenosis.

During his evaluation in our center, the ejection fraction was calculated as 48% and the basal, mid-basal posterior and anterior segments of the interventricular septum were hypokinetic. Emergent coronary angiography showed 100% stenosis in the left anterior descending artery and the stent previously placed was patent in the main circumflex artery, while 50% stenosis was observed in the second obtuse marginal artery, followed by 100% stenosis in the main circumflex artery (Figure 2). We decided to perform an elective CABG surgery as a treatment option for the patient, according to the results of the previous examinations.Preoperative written informed consent was obtained from the patient for open heart surgery.

Under general anesthesia, the patient received continuous esmolol infusion (50 µg/kg/min). The surgery was performed with off-pump beating heart technique and the heart was stabilized using Octopus(r) (Medtronic Inc, Minneapolis, USA) tissue stabilizers. The left internal mammary graft was anastomosed to the left anterior descending artery and a saphenous vein graft was anastomosed sequentially to the second obtuse marginal artery without any intraoperative complication. The patient was transferred to the cardiovascular intensive care unit under positive inotropic support (5 µg/kg/min Dopamine). The patient was, then, successfully extubated and discharged on the sixth day after the surgery.

## DISCUSSION

Moyamoya disease is a progressive, occlusive cerebrovascular disease without any known etiology which is mostly seen in the Northeast Asian countries^[^^1^^]^.

The disease is characterized by the occlusion or spontaneous bilateral stenosis of the terminal portion of the internal carotid artery^[^^1^^]^. Transient or permanent cerebral ischemic conditions are typically seen in the childhood, while hemorrhagic diseases are seen in the adulthood^[^^1^^]^. As a result, Moyamoya disease may present with distinct clinical presentations according to the age of the affected individual^[^^1^^]^.

To the best of our knowledge, there are three cases of Moyamoya disease operated with off-pump CABG technique. One of these cases was a 51-year-old Japanese female patient in whom angioplasty was performed for the left anterior descending artery stenosis, followed by elective left anterior mini thoracotomy and minimally invasive direct CABG due to the heart ischemia as assessed by the exercise stress test at 28 weeks of follow-up^[^^1^^]^. The other cases were a 56-year-old Korean female patient in whom off-pump three-vessel CABG was performed one year before the diagnosis of Moyamoya disease^[^^3^^]^ and a 48-year-old Japanese female patient in whom off-pump CABG was performed with the support of intra-aortic balloon pump (IABP) due to the left main coronary lesion^[^^4^^]^. Due to the low number of cases and limited intraoperative data, it is challenging to discuss the prevention methods for cerebral ischemia in Moyamoya disease. Prevention of cerebral ischemia in off-pump technique can be achieved by maintaining the systemic blood pressure over 80 mmHg. In addition, some authors reported the use of IABP to prevent cerebral diseases in patients with Moyamoya disease undergoing CABG^[^^4^^]^.

In the present case of Moyamoya disease, we believe off-pump two-vessel CABG provided a safe approach most probably by preventing CPB-related hypotensive cerebral ischemia. We think this type of management may provide effective prevention of intraoperative and postoperative complications.

**Table:** 

Authors' roles & responsibilities	
EC	Substantial contributions to the conception or design of the work; or the acquisition, analysis, or interpretation of data for the work; drafting the work or revising it critically for important intellectual content; final approval of the version to be published
LA	Substantial contributions to the conception or design of the work; or the acquisition, analysis, or interpretation of data for the work; final approval of the version to be published
UT	Drafting the work or revising it critically for important intellectual content; final approval of the version to be published
AT	Substantial contributions to the conception or design of the work; or the acquisition, analysis, or interpretation of data for the work; final approval of the version to be published
